# Dynamic changes in community structure and degradation performance of a bacterial consortium MMBC-1 during the subculturing revival reveal the potential decomposers of lignocellulose

**DOI:** 10.1186/s40643-022-00601-8

**Published:** 2022-10-22

**Authors:** Jingrong Zhu, Jiawen Liu, Weilin Li, Yunrui Ru, Di Sun, Cong Liu, Zongyun Li, Weijie Liu

**Affiliations:** 1 Jiangsu Key Laboratory of Phylogenomics & Comparative Genomics, School of Life Science, Jiangsu Normal University, No.101, Shanghai Road, Tongshan New District, Xuzhou, 221116 Jiangsu Province China; 2 Institutional Center for Shared Technologies and Facilities, Institute of Microbiology, Chinese Academy of Sciences, Beijing, 100101 China

**Keywords:** Taxonomy, Subculture, Cellulose, Hemicellulose, 16S rRNA gene

## Abstract

Bacterial consortium is an important source of lignocellulolytic strains, but it is still a challenge to distinguish the direct decomposers of lignocellulose from other bacteria in such a complex community. This study aims at addressing this issue by focusing on the dynamic changes in community structure and degradation activity of MMBC-1, an established and stable lignocellulolytic bacterial consortium, during its subculturing revival. MMBC-1 was cryopreserved with glycerol as a protective agent and then inoculated for revival. Its enzyme activities for degradation recovered to the maximum level after two rounds of subculturing. Correspondingly, the cellulose and hemicellulose in lignocellulosic carbon source were gradually decomposed during the revival. Meanwhile, the initial dominant bacteria represented by genus *Clostridium* were replaced by the bacteria belonging to *Lachnospira*, *Enterococcus*, *Bacillus*, *Haloimpatiens* genera and family Lachnospiraceae. However, only three high-abundance (> 1%) operational taxonomic units (OTUs) (*Lachnospira*, *Enterococcus* and *Haloimpatiens* genera) were suggested to directly engage in lignocellulose degradation according to correlation analysis. By comparison, many low-abundance OTUs, such as the ones belonging to *Flavonifractor* and *Anaerotruncus* genera, may play an important role in degradation. These findings showed the dramatic changes in community structure that occurred during the subculturing revival, and paved the way for the discovery of direct decomposers in a stable consortium.

**Graphical Abstract:**

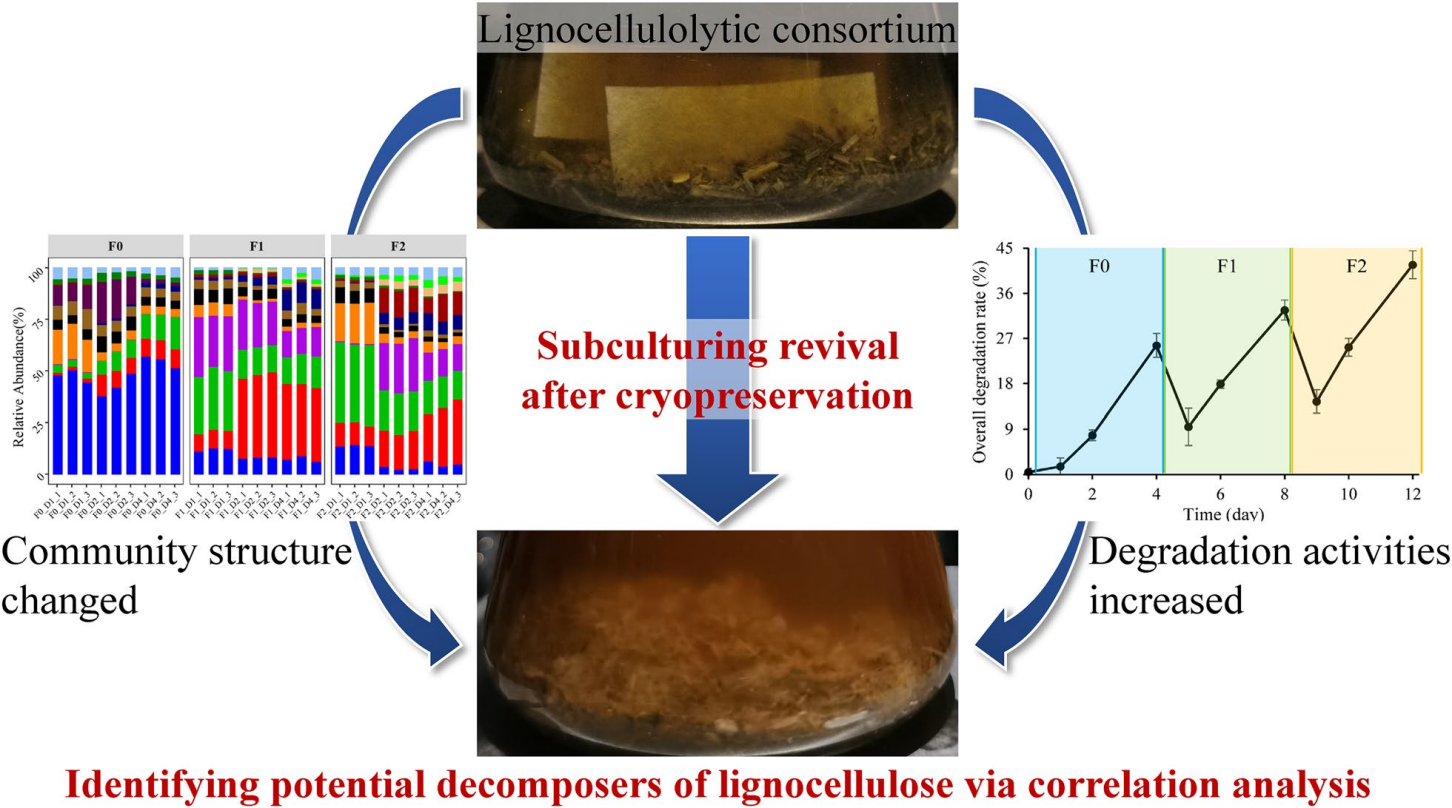

**Supplementary Information:**

The online version contains supplementary material available at 10.1186/s40643-022-00601-8.

## Introduction

Microorganisms with carbohydrate-active enzymes (CAZymes) are primarily responsible for the breakdown of lignocellulose, the richest renewable organic polymer in nature (Bomble et al. 2017). These microorganisms address industrial demands by producing CAZymes or high-value products such as biofuels, oligosaccharides, and ferulic acids from lignocellulosic biomass, in addition to their significant role in the global carbon cycle (Liu et al. 2022, 2019; Wang et al. 2020a). Nonetheless, the inefficiency of enzymatic deconstruction of lignocellulose with high recalcitrance jeopardizes bioconversion’s viability (Lopes et al. 2018). As a result, the search for microbes that produce effective CAZymes has long been prioritized.

A microbial consortium is a complex community in which multiple microorganisms synergistically perform metabolic processes, such as lignocellulose decomposition (Qian et al. 2020). Many investigations have focused on establishing effective microbial consortia, then isolating the microorganisms with high lignocellulose degradation activities from the consortia or cloning the genes of important CAZymes from their metagenomes (Zhang et al. 2016, 2021). Currently, the microbial consortia from the gastrointestinal tracts of herbivores and of phytophagous insects have been widely investigated, but the in vitro duplication of their efficacy in lignocellulose degradation is still a challenge (Li et al. 2022; Peng et al. 2021). The synthetic microbial consortia made up of a few strains are also appealing, but they have yet to overcome their inefficiency and instability. (Gilmore et al. 2019; Wang et al. 2021). Another frequent strategy to obtain microbial consortia is to enrich lignocellulolytic bacteria using raw biomass or plant polysaccharide as a carbon source. Many efficient consortia have been established utilizing environmental samples such as compost and soil samples (Diaz-Garcia et al. 2020; Lemos et al. 2017), and some of them can even generate the desired products straight from raw biomass, showing a promising prospect in the industrial application (Shahab et al. 2020).

During the enrichment, the activities of lignocellulolytic enzymes and the abundances of microorganisms involved in degradation both increase gradually (Cortes-Tolalpa et al. 2016; Diaz-Garcia et al. 2021). The correlation analysis between the degradation activities and the abundances of microorganisms during the enrichment can assist in identifying the primary microbial decomposers of lignocellulose in a consortium (Qiao et al. 2019). It is notable that many microorganisms play a role in forming an anaerobic environment, maintaining suitable pH, providing essential nutrients for the consortium rather than directly degrading lignocellulose (Ozkaya et al. 2017; Partanen et al. 2010; Sheng et al. 2016). Although these microorganisms are not directly responsible for lignocellulose degradation, they provide crucial circumstances for the real decomposers and are thus reserved and enriched in the consortium. The existence of these auxiliary microorganisms, on the other hand, would make it difficult to identify direct decomposers because these two types of microbes may share comparable dynamic changes in abundances throughout the consortium establishment process. Therefore, it is necessary to further distinguish the direct decomposer of lignocellulose from the other microorganisms in an established microbial consortium to obtain desired enzymes and strains.

After freeze preservation, a microbial consortium must be revived through subculturing (McClure et al. 2020). The original degrading activities of microbial consortia commonly fade away at first, then return with subculturing to their pre-cryopreservation levels. Therefore, the analysis of correlation between the degradation activities and bacterial abundances during revival can aid in the identification of the essential microorganisms for lignocellulose degradation in a stable microbial consortium. However, the succession of microbial communities during revival is still poorly understood.

In this study, a lignocellulolytic bacterial consortium, MMBC-1, was constructed using soil samples as inoculum and sweetpotato (*Ipomoea batatas*) straw as carbon source, and was then cryopreserved using glycerol as a protect agent. Subsequently, the dynamic changes of MMBC-1 in lignocellulose degradation, activities of lignocellulolytic enzymes and community composition during the subculturing revival were determined. Finally, the correlation between the bacterial abundances and the degradation activities was analyzed to investigate the potential roles of key bacteria. This study revealed the evolution of community structure during the subculturing revival, and identified the prospective bacteria that directly decomposing lignocellulose in MMBC-1.

## Materials and methods

### Establishment of lignocellulolytic bacterial consortium MMBC-1

The sample was collected from the saline-alkali soil in Daqing city, China, and inoculated into a 150-mL conical flask containing 100 mL of medium (5 g∙L^−1^ of tryptone, 5 g∙L^−1^ of NaCl, 2 g∙L^−1^ of yeast extract powders, 20 g∙L^−1^ of sweetpotato straw powders, 5 g∙L^−1^ of filter paper strip (3 cm * 1 cm) and 3 g∙L^−1^ of Na_2_CO_3_), where the Na_2_CO_3_ was used to create an alkali environment. The sweetpotato straw was collected from the farmland in Xuzhou city, China. Its cellulose, hemicellulose and lignin contents are 48.2%, 25.3% and 16.7%, respectively. The stationary culture was then carried out at 37 °C. Every 6 days, 10 mL of culture, including solids, was transferred into fresh mediums until the filter paper was broken. After that, the transfer was performed every 4 days until the filter paper could be completely decomposed within 4 days. The consortium culture was then mixed with 50% glycerol in an equal volume and cryopreserved at − 80 °C in a freezer for further use.

### Subculturing revival of MMBC-1

The consortium stock of 10 mL was inoculated into fresh medium and incubated at 37 °C. The culture of 10 mL was transferred into fresh medium every 4 days until the filter paper was completely decomposed. At 24, 48 and 96 h after inoculation and subculturing, three copies of the culture were collected to determine the community structure, lignocellulose degradation rate and enzymatic activities.

### Enzyme assays

The culture was filtered to obtain filtrate for determining enzymatic activities in the Na_2_HPO_4_–citric acid buffer (pH 6.5). Table 1 lists the substrates used in the assay of various enzymatic activities. The medium without inoculation was used as a control group.

**Table 1:** Substrates and reaction conditions for enzymatic assay

Enzyme type	Substrate	Substrate brand
Endo-β-1,4-glucanase	Carboxymethylcellulose, 1% (w/v)	Sinopharm, China
Endo-β-1,4-xylanase	Glucuronoxylan, 1% (w/v)	Megazyme, Ireland
Cellobiohydrolase	4-Nitrophenyl-β-D-cellobioside, 10 mM	Chemsynlab, China
β-Glucosidase	4-Nitrophenyl β-D-glucopyranoside, 10 mM	Chemsynlab, China
β-Xylosidase	4-Nitrophenyl β-D-xylopyranoside, 10 mM	TCI, Japan
α-L-Arabinofuranosidase	4-Nitrophenyl α-L-arabinofuranoside, 10 mM	Chemsynlab, China

To determine the activities of endo-β-1,4-glucanase and endo-β-1,4-xylanase, 50 μL of dilute filtrate was mixed with 100 μL of substrate solution, and the mixture was immediately incubated at 50 °C for 20 min. Subsequently, 200 μL of 3,5-dinitrosalicylic acid was added into the mixture, which was then incubated for 5 min in a boiling water bath (Miller 1959). After cooling, 1 mL of deionized water was added. The supernatant was collected after centrifugation, and its absorbance at 520 nm was finally determined to calculate the enzymatic activity.

To determine the activities of cellobiohydrolase and three glycosidases (β-glucosidase, β-xylosidase, α-L-arabinofuranosidase), 100 μL of dilute filtrate was mixed with 100 μL of substrate solution, and the mixture was immediately incubated at 50 °C for 10 min. Subsequently, 100 μL of Na_2_CO_3_ solution (1.0 M) were added into the mixture to terminate the reaction. After that, 300 μL of deionized water was added, and the absorbance at 405 nm was determined to calculate the enzymatic activity.

The dilute filtrate of 150 μL were sampled to determine the concentration of reducing sugar in the medium with 3,5-dinitrosalicylic acid as described above. To determine the protein concentration in the medium, 50 μL of dilute filtrate were mixed with 1 mL of Bradford reagent (Bio-Rad, USA). After stationary reaction at room temperature for 5 min, the absorbance at 595 nm was determined to calculate the protein concentration.

### Determination of lignocellulose degradation rate

The culture was filtered to obtain the residues to determine the degradation rate of sweetpotato straw. The residues were pulverized using a mortar and liquid nitrogen. After freeze drying, the residues were weighed to calculate their weight loss after culture, thereby determining the overall degradation rate. Then, 0.5 g of residue powders was sampled to determine the contents of cellulose, hemicellulose and lignin based on an established method (Sluiter et al. 2008). In brief, the powders were acidolysis in 72% and 4% of sulfuric acid successively. Subsequently, the liquid and the residue were separately collected by filtration. The liquid of 20 μL was sampled to quantify the cellulose and hemicellulose contents by measuring the monosaccharide concentrations using high performance liquid chromatography equipped with a Shodex Sugar SP0810 column. Ultrapure water was employed as the mobile phase with a flow rate of 1.0 mL/min at 80 °C and the monosaccharides were detected using a refractive index detector (Shodex RI-201H). The residue was ashed for 6 h at 600 °C, and the weight loss after ashing was calculated to determine the lignin content.

### DNA extraction and sequencing data analysis

The culture (10 mL) was sampled and the cells were then collected by centrifugation at 4 °C for 10 min with a relative centrifugal force of 10,000 g. The metagenomic DNA of MMBC-1 was extracted using a FastDNA Spin Kit for Soil (MP Biomedicals, USA) according to manufacturer’s instructions. The V3–V4 sequences of 16S rRNA genes were amplified using 338F/806R universal primers and sequenced on an Illumina MiSeq platform (Majorbio Company, China). The raw data were trimmed via quality filter and analyzed using the QIIME software (Caporaso et al. 2010). The primer sequences and low-quality sequences with primer mismatch, ambiguous bases or a length short than 100 bp were discarded. Afterwards, the sequences were clustered into operational taxonomic units (OTUs) at 97% sequence similarity and the chimeric sequences were removed using USEARCH version 9.2 (Edgar 2013). The most abundant sequence in each OTU was retrieved as the representative sequence, which was further classified taxonomically against the Silva128 database (Pruesse et al. 2007).

### Bioinformatics and statistical analysis

The redundancy analysis (RDA) was employed to investigate the relationship between bacterial community and environment parameters. In brief, the data were firstly Hellinger transformed and the variance inflation factors (VIF) of environmental parameters were then calculated. To avoid the multicollinearity of environment parameters, only the environment parameters with VIF < 20 were selected for RDA. During RDA, Monte Carlo permutation test (with 999 permutations) was performed for the forward selection of environment parameters. Statistical analyses were carried out using vegan package via R 4.0.3.

Pearson correlation coefficients between the relative abundance of OTUs and environmental parameters were calculated using SPSS version 18.0, and *P*-values under 0.05 (*P* < 0.05) were considered statistically significant.

## Results and discussion

### Consortium establishment

To establish a stable bacterial consortium, the alkaline soil samples were employed as a microbial source to enrich the bacteria with lignocellulolytic activities. After 10 times of subculturing, the filter papers in medium were disintegrated within 6 days. After another 5 times of subculturing, the filter papers were completely decomposed within 4 days. Such degradation activity remained stable thereafter. The established consortium, MMBC-1, was cryopreserved at − 80 °C using glycerol as a protective agent for further use. MMBC-1 possessed a unique characteristic of tolerating the alkaline environment, suggesting that saline-alkali soil is a good source of extreme microorganisms. Many monocotyledonous crop straws like wheat and rice have been used as carbon sources for the enrichment of lignocellulolytic bacteria (Cortes-Tolalpa et al. 2018; Zheng et al. 2020), whereas dicots, whose hemicellulose structure and content are different from monocotyledonous crop straws, have received less attention (Ebringerová and Heinze 2000). In this study, the straw of dicotyledonous sweetpotato, the sixth most important food crop in the world (Yang et al. 2019), was used as a carbon source. The established consortium was able to rapidly decompose the filter papers at moderate temperature (37 °C), suggesting such straw had a good potential for enriching effective lignocellulolytic bacteria.

**Fig. 1:**
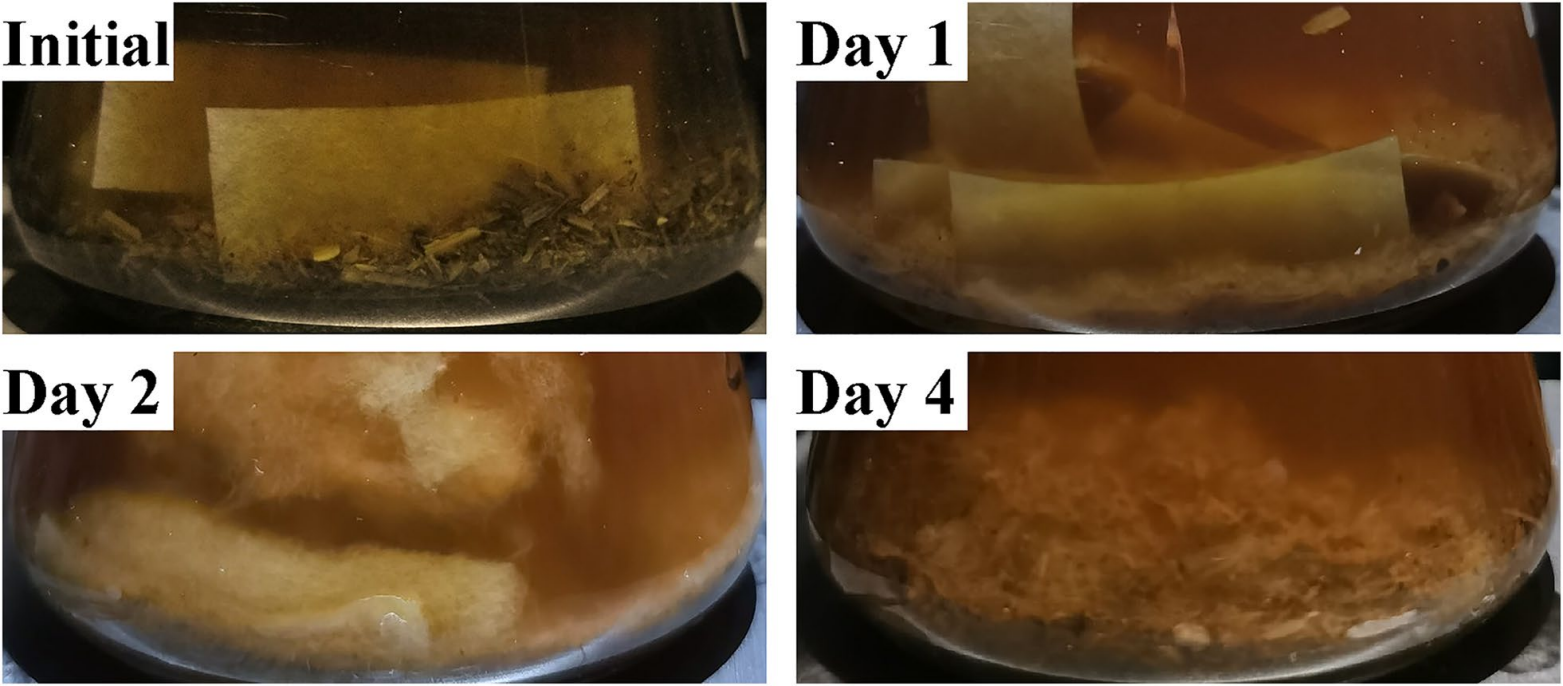
Degradation of filter papers during consortium revival

Like most reported lignocellulolytic bacterial consortia, MMBC-1 temporarily lost degradation activity after the transfer from glycerol stock to fresh medium (F0). After the first subculturing (F1), the filter papers in medium began to swell, which is a sign of breakdown. After the second subculturing (F2), the filter papers were totally decomposed within 4 days, demonstrating that the degradation activity of MMBC-1 had recovered to the maximum level (Fig. 1). The freezing process may result in the inactivation of some bacteria, thereby destroying the community structure. After several times of subculturing, the community structure returned to the original state with high lignocellulose degradation efficiency. However, the microbial community succession during subculturing revival is poorly understood.

### Dynamic changes in components of the medium

**Fig. 2:**
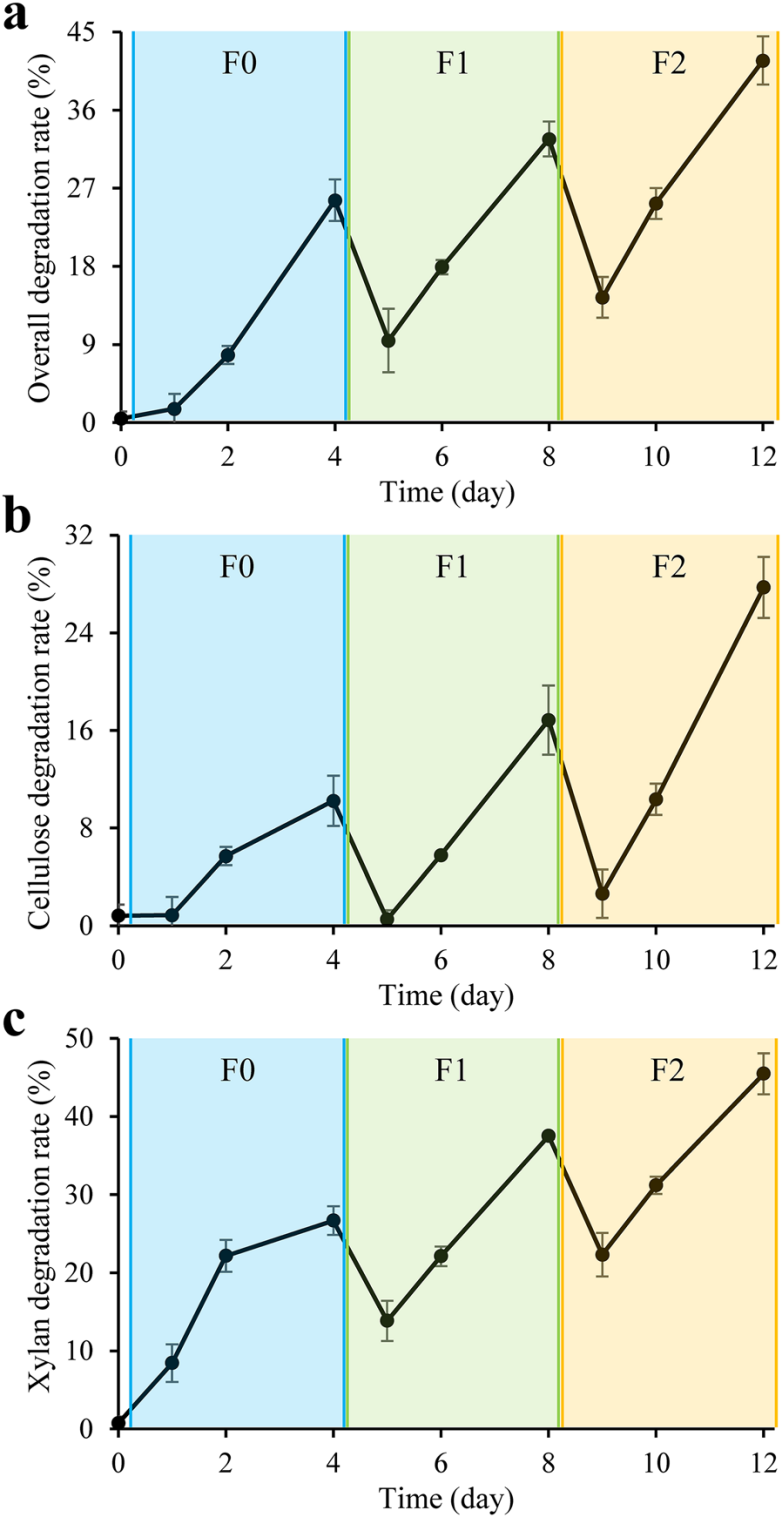
Degradation rates of the **A** entire carbon source, **B** cellulose and **C** hemicellulose during revival

To reveal the degradation activity changes of MMBC-1 during the revival, the lignocellulose degradation rate was determined. The results indicated that MMBC-1 mainly decomposed the polysaccharides (cellulose and hemicellulose) of lignocellulose for utilization, but hardly degraded the lignin. Although the filter papers in medium did not visually change at F0D4, the overall degradation rate of carbon source reached 25.6%, which account for 61.4% of that in end of the revival (F2D4) (Fig. 2A). The cellulose degradation rate was 10.2% at F0D4, which only account for 36.8% of that at F2D4 (Fig. 2B). By comparison, the hemicellulose degradation rate was 26.7% at F0D4, which only account for 58.7% of that at F2D4 (Fig. 2C). In the early stage of every subculturing, the cellulose was slightly decomposed (0.9%, 0.5% and 2.6% at F0D1, F1D1 and F2D1, respectively), while much more hemicellulose was degraded at the same time (8.4%, 13.8% and 22.3% at F0D1, F1D1 and F2D1, respectively). Therefore, the degradation of hemicellulose took precedence over that of cellulose, and the decomposition of cellulose was accelerated in the late subculturing and revival.

**Fig. 3:**
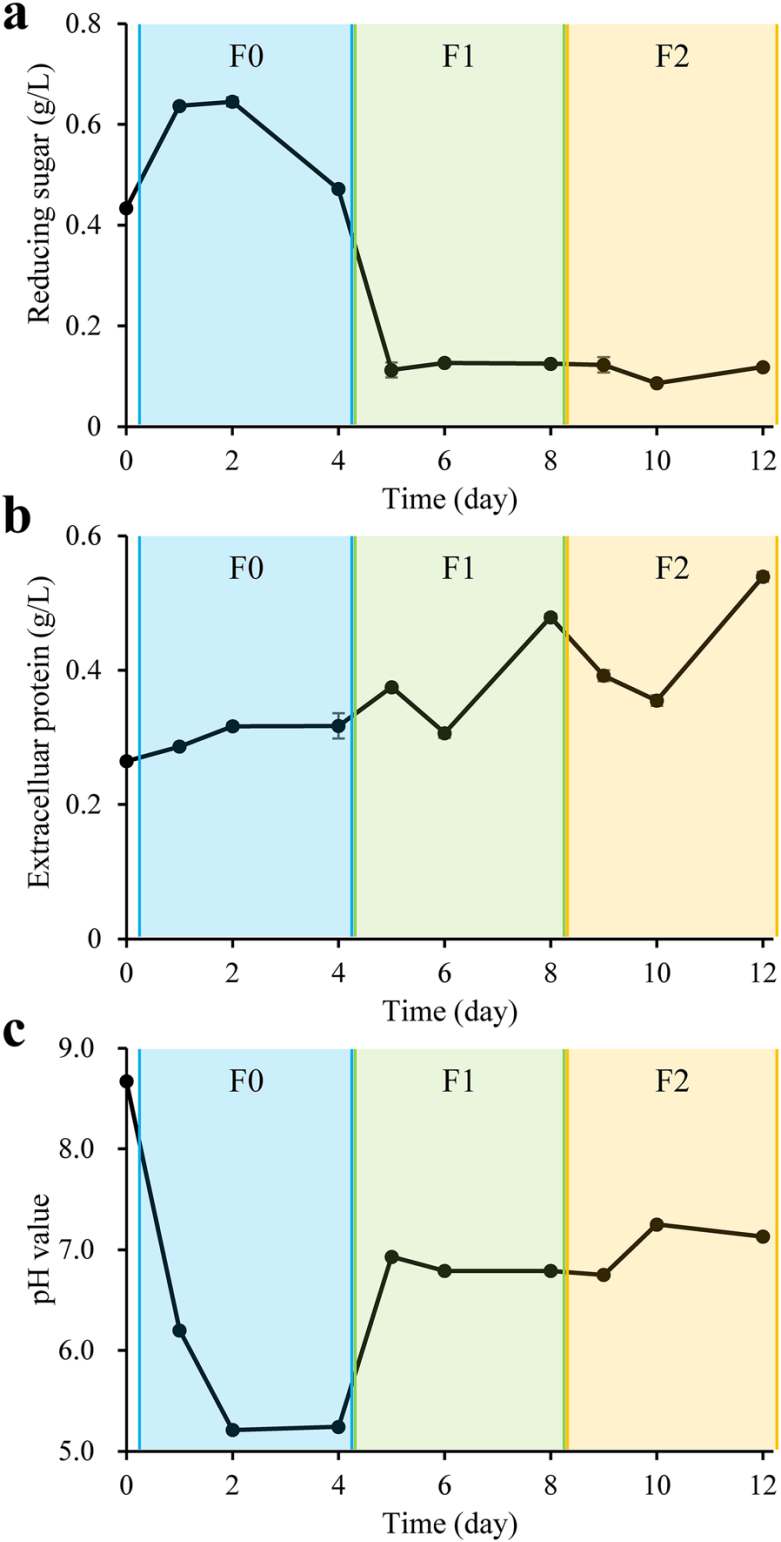
Evolutions of **A** reducing sugar, **B** extracellular protein and **C** pH of the medium

The concentration of reducing sugar in the medium was 0.43 mg/mL at the beginning, which further increased during the culture in F0. After the first subculturing, however, the concentration decreased rapidly and stabilized around 0.12 mg/mL (Fig. 3A), suggesting the monosaccharides and oligosaccharides resulted from lignocellulose degradation were used by bacteria quickly. The protein concentration in the medium continually increased from initial 0.26 mg/mL to 0.54 mg/mL (Fig. 3B), which was possibly due to the extracellular enzymes secreted by the bacteria. The pH decreased from an initial value of 8.7 to 5.2 at F0D4, which returned to the value around 7.0 whereafter (Fig. 3C). Although MMBC-1 was established using alkaline soil and alkaline medium, the lignocellulose degradation by MMBC-1 seemed to require a neutral environment.

### Dynamic changes in enzymatic activities

**Fig. 4:**
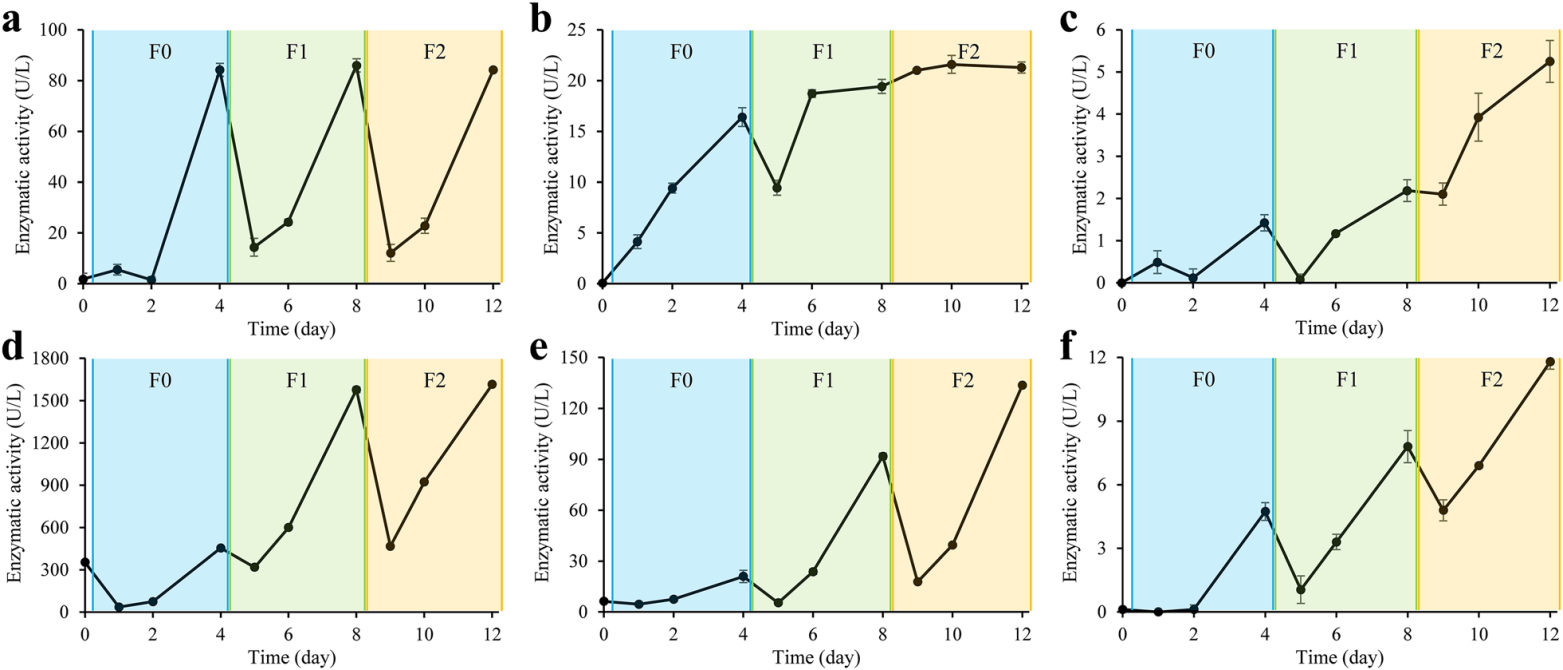
Dynamic changes in cellulase and hemicellulase activities during revival. **A** Endo-β-1,4-glucanase activity, **B** β-glucosidase activity, **C** cellobiohydrolase activity, **D** endo-β-1,4-xylanase activity, **E** α-L-arabinofuranosidase activity, **F** β-xylosidase activity

The degradation was accomplished by some CAZymes, so the activities of primary cellulases and hemicellulases were determined during the revival. As shown in Fig. 4A, the endo-β-1,4-glucanase activity at F0D4 surprisingly increased to the level of late revival (F2D4). The β-glucosidase activity also increased quickly and reached 16.4 U/L at F0D4, accounting for 77% of that at F2D4 (Fig. 4B). Nevertheless, the cellobiohydrolase activity was low in F0 and F1 (Fig. 4C). Therefore, the initial cellulose degradation rate may be limited by the low cellobiohydrolase activity (Fig. 2B).

The endo-β-1,4-xylanase activity was low in F0, which reached 455 U/L at F0D4, accounting for only 28% of that at F2D4. After the first subculturing, the endo-β-1,4-xylanase activity recovered rapidly to the maximum level (Fig. 4D). The α-L-arabinofuranosidase activity was also low in F0, reaching 21 U/L at F0D4 and accounting for just 16% of that at F2D4. At F1D4, its activity recovered to 69% of the maximum level (Fig. 4E). The β-xylosidase activity gradually increased to 11.8 U/L during the revival (Fig. 4F). The recovery of hemicellulase activities was slower than that of cellulases, but the degradation of hemicellulose took precedence over that of cellulose in the early stages of every subculturing and of the entire revival (Fig. 2B, C). It could be interpreted as the fact that cellulose is embedded in hemicellulose which has a higher enzyme accessibility (Carpita and McCann 2020). The endo-β-1,4-glucanase and endo-β-1,4-xylanase activities rapidly increased to their maximum levels, whereas the others completely recovered at F2D4. Therefore, it can be speculated that the bacteria producing endo-β-1,4-glucanase and endo-β-1,4-xylanase rejuvenated in F1 or even F0, while the bacteria that produce the other enzymes gradually recovered until the end of revival.

### Taxonomic changes in MMBC-1 during subculturing revival

**Fig. 5:**
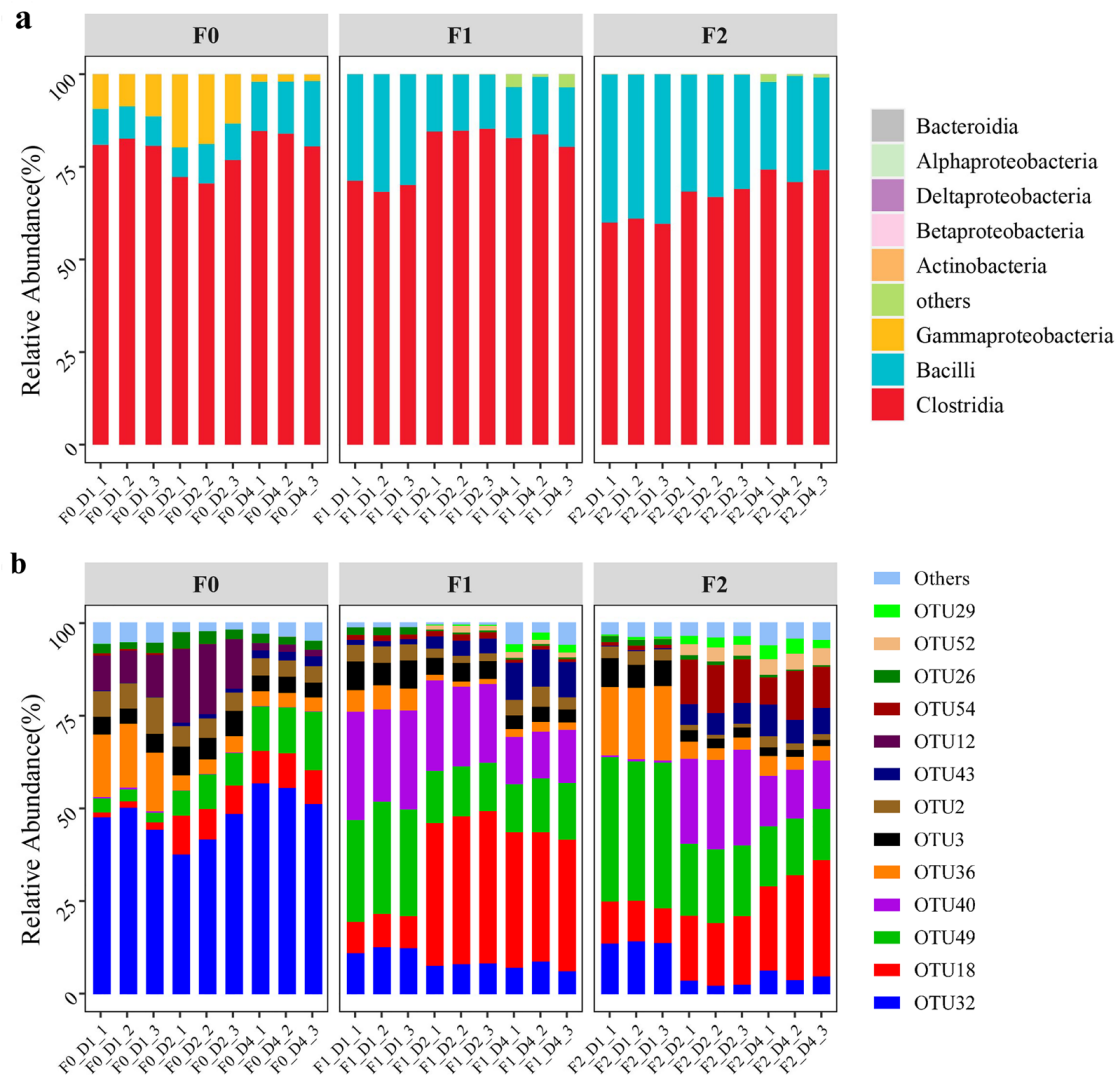
Succession of the microbial community at **A** class level and **B** OTU level during revival

To reveal the taxonomic changes during the subculturing revival, MMBC-1 was sampled at 24 h (D1), 48 h (D2) and 96 h (D4) after every culture transfer to determine its community structure. MMBC-1 contained five bacterial phyla, among which Firmicutes was the most dominant phylum with an average abundance of over 99.90% (Additional file 2: Table S1). There were 13 bacterial classes identified in MMBC-1, among which Clostridia, Bacilli and Gammaproteobacteria were the dominant in F0 (Fig. 5A). After subculturing, however, the abundance of Gammaproteobacteria decreased from 9.75% at F0D1 to less than 0.01% at F2D4 and meanwhile, the abundance of Bacilli increased from 8.83% to 25.75%. At the end of revival, Clostridia and Bacilli became the dominant classes, making up more than 99% of the consortium together. As the degradation activity of MMBC-1 did not recover until F2, these two bacterial classes were presumably important to lignocellulose decomposition.

In total, 54 OTUs were identified in the entire revival process, with 13 of them being high-abundance (> 1%) (Fig. 5B and Table 2). In F0, the abundance of OTU32 belonging to genus *Clostridium* was the highest, accounting for about half of the consortium. Because glycerol was used as a cryoprotectant, the medium of F0 after inoculation contained a high proportion of glycerol (approximately 2.27%). Furthermore, the OTU32 is closely matched to *Clostridium butyricum* that is well-known for utilizing glycerol (Wang et al. 2020b). Therefore, OTU32 may be stimulated by such carbon source in F0. The OTU36, another OTU belonging to genus *Clostridium*, and OTU12 belonging to genus *Pseudomonas* were also dominant in the early stage of F0, accounting for 16.5% and 9.7% at F0D1, respectively. Nevertheless, the abundances of OTU36 and OTU12, respectively, reduced to 3.8% and 1.9% after 3 days of culture (F0D4). Subculturing showed a profound impact on the community structure. Specifically, the abundance of OTU32 (genus *Clostridium*) decreased from 54.6% at F0D4 to 5.3% at F2D4. Meanwhile, the abundances of OTU18, OTU54, OTU43, OTU29 and OTU52, which were the five most enriched OTUs, increased 16-fold, 20-fold, 55-fold, 69-fold and 357-fold during the entire revival (from F0D1 to F2D4), respectively (Additional file 1: Figure S1). Some of these enriched OTUs, such as OTU18 (genus *Lachnospira*), OTU54 (genus *Bacillus*) and OTU52 (genus *Flavonifractor*), were previously confirmed or suggested to participate in the decomposition of plant polysaccharides like xylan and pectin (Karuppiah et al. 2021; Moro Cantu-Jungles et al. 2019; Yin et al. 2017), while the roles of OTU43 (genus *Haloimpatiens*) and OTU29 (genus *Anaerotruncus*) were currently unknown. Present investigation demonstrated a positive correlation between the abundance of these two OTUs and the degradation activity of MMBC-1, indicating potential contributions of *Haloimpatiens* and *Anaerotruncus* genera to the decomposition of lignocellulose for the 1st time. At F2D4, OTU18, OTU49, OTU40, OTU54 and OTU43 became dominant bacteria, together accounting for 73.2% (Fig. 5B). Among them, the OTU18 (genus *Lachnospira*), OTU54 (genus *Bacillus*) and OTU43 (genus *Haloimpatiens*) were also the most enriched bacteria as mentioned above. Genus *Enterococcus* (OTU49), which is common in the bacterial consortia from intestinal tracts of herbivores, was perceived as the promoter of lignocellulose degradation (Jin et al. 2021; Li et al. 2021), and the bacteria belonging to family Lachnospiraceae (OTU40) were also representative decomposers of lignocellulose (Suksong et al. 2019), suggesting that they may be conducive to the degradation activity of MMBC-1.

**Table 2:** Taxonomic affiliations of the most abundant (> 1%) OTUs

OTU	Taxon at genus level	Closest valid taxon (Id. %)^b^
OTU2	*Romboutsia*	*Romboutsia lituseburensis* (98.58)
OTU3	*Terrisporobacter*	*Terrisporobacter petrolearius* (99.06)
OTU12	*Pseudomonas*	*Pseudomonas songnenensis* (99.33)
OTU18	*Lachnospira*	*Lactobacillus rogosae* (96.00)
OTU26	*Asaccharospora*	*Asaccharospora irregularis* (99.06)
OTU29	*Anaerotruncus*	*Anaerotruncus rubiinfantis* (97.17)
OTU32	*Clostridium*	*Clostridium butyricum* (99.29)
OTU36	*Clostridium*	*Clostridium tertium* (99.06)
OTU40	Unc. Lachnospiraceae^a^	*Variimorphobacter saccharofermentans* (97.41)
OTU43	*Haloimpatiens*	*Haloimpatiens lingqiaonensis* (99.06)
OTU49	*Enterococcus*	*Enterococcus casseliflavus* (99.33)
OTU52	*Flavonifractor*	*Flavonifractor plautii* (99.30)
OTU54	*Bacillus*	*Bacillus yapensis* (99.11)

^a^*Unc* unclassified

^b^The identity was determined by BLAST in NCBI

The community structure of MMBC-1 greatly changed during the revival despite being a stable consortium. Specifically, the dominant bacteria in F0 like OTU32 (genus *Clostridium*), OTU36 (genus *Clostridium*) and OTU12 (genus *Pseudomonas*) were replaced by the bacteria that can decompose lignocellulose, including OTU18 (genus *Lachnospira*), OTU49 (genus *Enterococcus*), OTU40 (family Lachnospiraceae) and OTU54 (genus *Bacillus*). It is noteworthy that OTU18, OTU49 and OTU40 were already dominant in F1, but the degradation activity of MMBC-1 did not recover to the maximum level until the abundance of some other bacteria including OTU52 (genus *Flavonifractor*) and OTU54 (genus *Bacillus*) greatly increased in F2, suggesting their significant roles in lignocellulose degradation.

### Relationship between degradation activity and OTU abundance

**Fig. 6:**
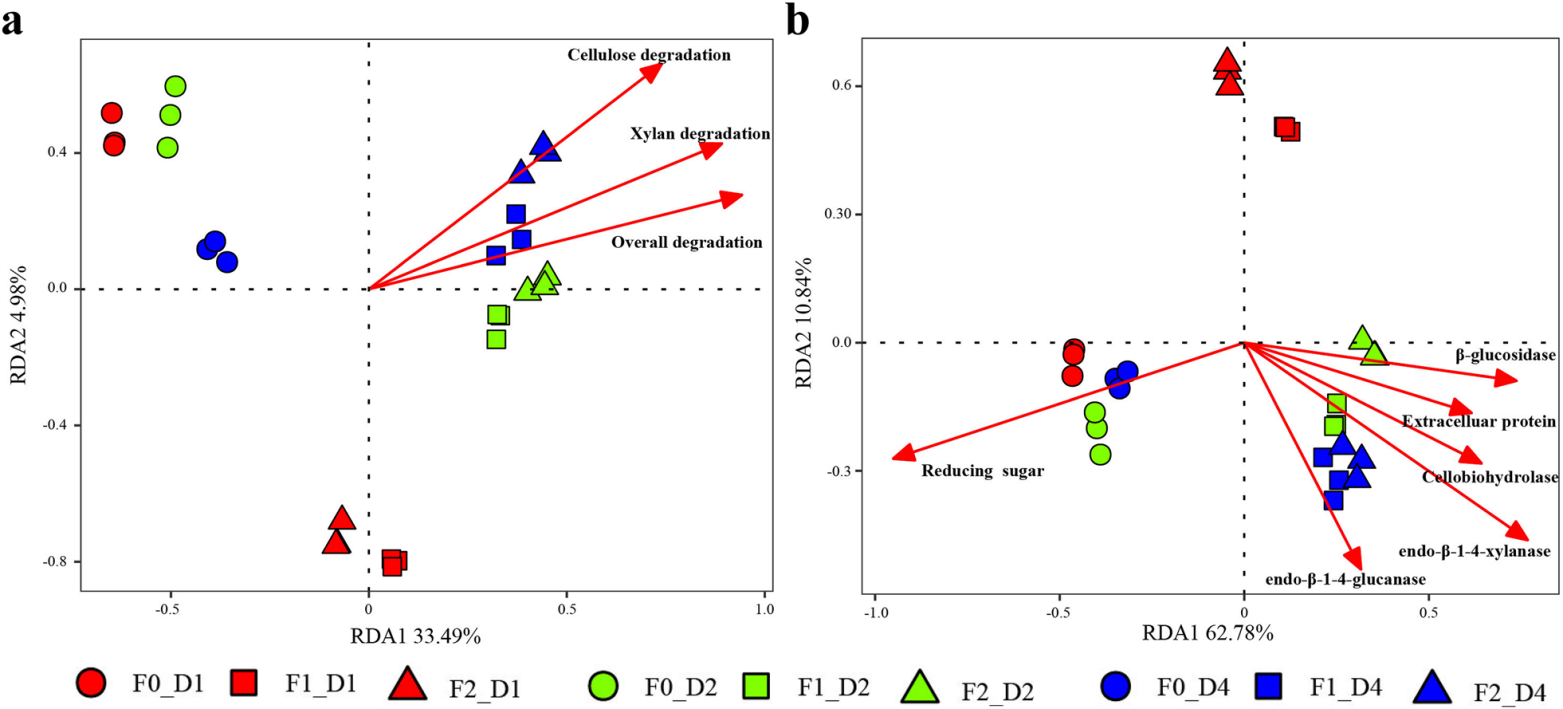
Redundancy analysis of the relationship between degradation activities and bacterial community composition. **A** The relationship between degradation rates of carbon source and community composition. **B** The relationship between environmental parameters and community composition

RDA was applied to assess the relationships between bacterial community structure and degradation activities of MMBC-1 (Fig. 6). A Monte Carlo permutation test within RDA showed that the community structure of MMBC-1 significantly correlated with the degradation rates of carbon source (overall degradation, cellulose degradation, xylan degradation), cellulase and hemicellulase activities (endo-β-1–4-glucanase, cellobiohydrolase, β-glucosidase, endo-β-1–4-xylanase), reducing sugar concentration and extracellular protein concentration (*P* < 0.05, see Additional file 3: Table S2 and Additional file 4: Table S3 for details). The community structures of MMBC-1 at F1D2, F1D4, F2D2 and F2D4 were most related to higher lignocellulose degradation rates as well as higher activities of endo-β-1-4-glucanase, cellobiohydrolase, β-glucosidase, endo-β-1-4-xylanase, indicating that the bacterial consortium evolved in a direction favorable to lignocellulose decomposition during revival.

**Fig. 7:**
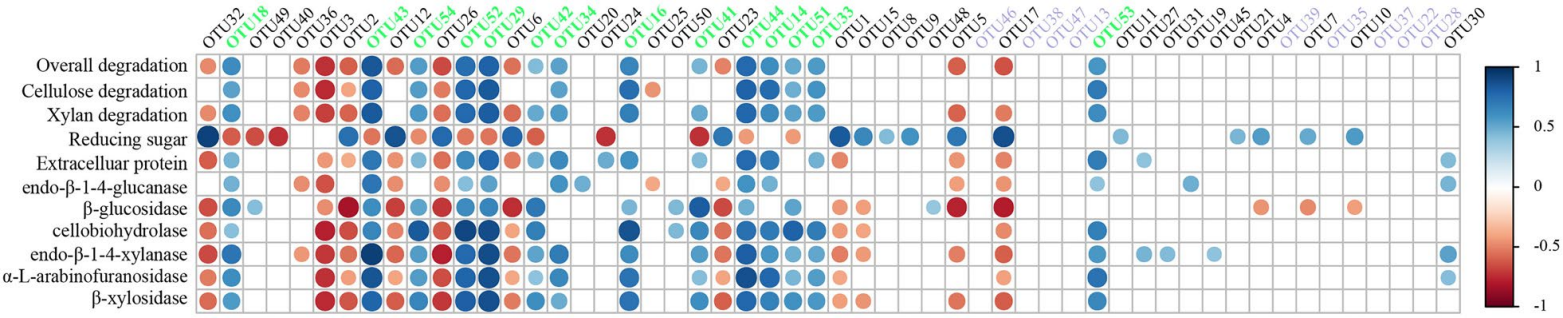
Pearson correlation between OTU abundances and degradation activities. Blue and red circles indicate significant (*P* < 0.05) positive and negative correlations, respectively

To further identify the potential bacteria participating in the degradation, Pearson correlations between OTU abundances and degradation activities were analyzed (Fig. 7). There were 14 OTUs that positively correlated with the overall degradation rate of carbon source (*P* < 0.05, see Additional file 5: Table S4 for details), including three dominant OTUs (OTU18, OTU43 and OTU54) and 11 low-abundance (< 1%) OTUs at F2D4. It is notable that other high-abundance OTUs (Table 2), including two dominant OTUs (OTU40 and OTU49), positively correlate with neither lignocellulose degradation rates nor most enzymatic activities, suggesting that these bacteria had a limited contribution to degradation despite their high abundance. By comparison, a strong positive correlation between the degradation rates and the five most enriched OTUs during the revival (OTU18, OTU54, OTU43, OTU29 and OTU52) was observed, suggesting that the increment in abundance, rather than the final abundance, is a better indicator of the contribution to degradation. In addition, it is unexpected that so many low-abundance OTUs positively correlated with degradation activities. For examples, the low-abundance bacteria belonging to family Veillonellaceae (OTU33 and OTU34), family Lachnospiraceae (OTU14) and genus *Lachnoclostridium* (OTU16) were reported to contribute to the plant fiber degradation (Cerisy et al. 2017; Vahidi et al. 2021); OTU41 (genus *Faecalicatena*) and OTU42 (genus *Clostridium*) showed positive correlation with xylan degradation rate but not cellulose degradation rate, suggesting their preference of decomposing hemicellulose; OTU44 belonging to genus *Hydrogenoanaerobacterium* was also highly positively correlated with the overall degradation rate, suggesting its beneficial role in lignocellulose decomposition which is previously unknown. These findings suggested that many non-dominant bacteria that were enriched during the revival conduced a lot to the lignocellulose degradation besides certain dominant ones.

With the development of sequencing technology, metagenomics has become the most popular strategy for consortium research (Carlos et al. 2018). Although this method can directly display nearly all of the lignocellulolytic enzyme genes of a microbial consortium, the selection of efficient ones is still challenging. It is worth noting that the lignocellulose degradation is accomplished not by all the enzymes of a consortium, but by the most effective ones in a synergistic way (Tuncil et al. 2017). Therefore, further identifying the genuine decomposers is necessary. Present study showed that analyzing taxonomic succession during revival is a feasible way to identify the bacteria that may directly contribute to lignocellulose degradation, which would provide a theoretical basis for the development of efficient lignocellulosic enzymes. Moreover, these results suggest not only the contribution to lignocellulose degradation of certain dominant bacteria, but also an important role of some low-abundance ones. In addition, the contribution to lignocellulose degradations of some bacteria like *Anaerotruncus* (OTU29), *Haloimpatiens* (OTU43) and *Hydrogenoanaerobacterium* (OTU44) genera were suggested for the first time here. All these bacteria are expected to become the treasuries of novel lignocellulolytic enzymes in the future.

## Conclusions

During the subculturing revival of bacterial consortium MMBC-1, its degradation activities gradually increased and its community structure underwent tremendous changes where the lignocellulolytic bacteria became the dominant. Correlation analysis suggested that only a few dominant bacteria were directly involved in lignocellulose degradation while, surprisingly, many low-abundance bacteria contributed significantly. These results showed that studying on revival is a feasible way to identify the potential decomposers of lignocellulose in an established consortium, which could pave the way for the discovery of valuable strain and gene resources.

### Supplementary Information

**Additional file 1**: **Figure S1**. The evolution of high-abundance (>1%) OTUs during the revival.

**Additional file 2**: **Table S1**. The classification and abundance of all the OTUs.

**Additional file 3**: **Table S2**. The Monte Carlo permutation test within RDA between bacterial community composition and degradation rates.

**Additional file 4**: **Table S3**. The Monte Carlo permutation test within RDA between bacterial community composition and environmental parameters.

**Additional file 5**: **Table S4**. Pearson correlations between OTU abundances and degradation activities.

## Data Availability

The datasets generated during the current study are available in the Figshare repository (https://doi.org/10.6084/m9.figshare.20238807.v1).

## Abbreviations

CAZyme: Carbohydrate-active enzyme
OTU: Operational taxonomic unit
RDA: Redundancy analysis
