# Supplementary material for: Dynamic changes in community structure and degradation performance of a bacterial consortium MMBC-1 during the subculturing revival reveal the potential decomposers of lignocellulose
Source: Bioresour Bioprocess. 2022 Oct 22;9(1):110. doi: 10.1186/s40643-022-00601-8 (PMC10991580; doi:10.1186/s40643-022-00601-8)
Supplement: Supplementary file 3 — Additional file 3: Table S2. The Monte Carlo permutation test within RDA between bacterial community composition and degradation rates. [file 40643_2022_601_MOESM3_ESM.docx]

**Table S1 Monte Carlo permutation test within redundancy analysis between bacterial community composition and degradation rates**

|  | r^2^ | Pr(>r) |
| --- | --- | --- |
| Overall degradation | 0.6244 | 0.001*** |
| Cellulose degradation | 0.6455 | 0.001*** |
| Xylan degradation | 0.6484 | 0.001*** |
