# Supplementary material for: Dynamic changes in community structure and degradation performance of a bacterial consortium MMBC-1 during the subculturing revival reveal the potential decomposers of lignocellulose
Source: Bioresour Bioprocess. 2022 Oct 22;9(1):110. doi: 10.1186/s40643-022-00601-8 (PMC10991580; doi:10.1186/s40643-022-00601-8)
Supplement: Supplementary file 4 — Additional file 4: Table S3. The Monte Carlo permutation test within RDA between bacterial community composition and environmental parameters. [file 40643_2022_601_MOESM4_ESM.docx]

**Table S2 Monte Carlo permutation test within redundancy analysis between bacterial community composition and degradation activities**

|  | r^2^ | Pr(>r) |
| --- | --- | --- |
| Endo-β-1-4-glucanase | 0.3233 | 0.013* |
| cellobiohydrolase | 0.4501 | 0.001*** |
| β-glucosidase | 0.5264 | 0.001*** |
| Endo-β-1-4-xylanase | 0.7188 | 0.001*** |
| Reducing sugar | 0.9681 | 0.001*** |
| Extracelluar protein | 0.3800 | 0.006** |
